# The Significance of High-Density Lipoprotein-Derived Inflammatory Parameters in Atypical Parkinsonisms—Pilot Study

**DOI:** 10.3390/jcm14072212

**Published:** 2025-03-24

**Authors:** Piotr Alster, Bartosz Migda, Natalia Madetko-Alster

**Affiliations:** 1 Department of Neurology, Medical University of Warsaw, Kondratowicza 8, 03-242 Warsaw, Poland; natalia.madetko@wum.edu.pl; 2 Diagnostic Ultrasound Lab, Department of Pediatric Radiology, Medical University of Warsaw, 03-242 Warsaw, Poland; bartosz.migda@wum.edu.pl

**Keywords:** atypical parkinsonism, PSP, MSA, neurodegeneration, neuroinflammation

## Abstract

**Background/Objectives:** Atypical parkinsonisms are a group of diseases with significant obstacles in the context of efficient methods of examination and understanding pathomechanisms. This is associated with the overlaps in clinical manifestation. One of the hypotheses regarding the mechanism leading to neurodegeneration in this group is related to inflammation. **Methods:** Authors examined 18 patients with Multiple System Atrophy—Parkinsonism Predominant (MSA-P), 15 with Progressive Supranuclear Palsy—Richardson’s Syndrome (PSP-RS) and 15 with PSP—Parkinsonism Predominant (PSP-P) (disease duration: 3–6 years) using neutrophil-to-lymphocyte-ratio, platelet-to-lymphocyte ratio and high-density lipoprotein (HDL)-derived inflammatory ratios, e.g., neutrophil to high-density lipoprotein cholesterol ratio (NHR), lymphocyte to high-density lipoprotein cholesterol ratio (LHR) and platelet to high-density lipoprotein cholesterol ratio (PHR). The potential differences between the groups were examined using one-way ANOVA, with Tukey’s HSD test. **Results**: The comparison revealed significant differences between PSP-RS and MSA-P in NHR (*p* = 0.0224). The levels of the parameters were more increased in MSA-P. No other significant differences were found. **Conclusions:** The possible significance of HDL in the context of brain–blood barrier permeability is a repeatedly highlighted feature of neurodegenerative diseases. The outcome of this pilot study may suggest that the evaluation of inflammatory processes should be performed with the indication of subtypes of PSP, as the character of pathomechanisms likely differs.

## 1. Introduction

Atypical parkinsonisms are a heterogeneous group of diseases with diverse clinical manifestations. Among the diseases in this group, synucleinopathies such as Multiple System Atrophy (MSA) and tauopathies such as Progressive Supranuclear Palsy (PSP) and Corticobasal Degeneration (CBD) can be mentioned. The entities are characterized by pronounced progression of parkinsonism with falls, poor response to levodopa treatment and multiple non-motor features. In MSA, the significant feature is associated with dysautonomia; in PSP and CBD, apart from akinesia, language and cognitive impairment, the clinical manifestations differ depending on the subtype [1,2,3]. The criteria for diagnosis of PSP released in 2017 indicated multiple subtypes of PSP; however, up to 90% of all PSP cases are linked with two main subtypes: PSP—Richardson’s syndrome (PSP-RS) and PSP—Parkinsonism Predominant (PSP-P). In MSA, the criteria indicate a term of clinically established disease. In both cases, the definite diagnosis is based on neuropathological examination, which leads to the fact that it is not possible to provide it in vivo. The diagnostic tools enabling examination of atypical parkinsonisms, e.g., neuroimaging—magnetic resonance imaging and positron emission tomography, provide supplementary arguments in favor of certain diagnoses rather than specific differentiation. Evolving interest is linked with the area of searching for mechanisms leading to neurodegeneration in atypical parkinsonisms. Among theories regarding the pathomechanisms of PSP and MSA, we can mention neuroinflammation, oxidative stress and vascular factors, among others. The role of neuroinflammation is relatively briefly described; moreover, it is not specified whether the processes are a cause or consequence of neurodegeneration [4]. Additionally, growing interest is associated with the role of lipid profile alterations in the pathogenesis of neurodegenerative diseases [5]. Apolipoprotein A-I dysregulation is associated with blood–brain barrier dysfunction, oxidative stress and inflammation, which are elements of Parkinson’s disease and Alzheimer’s disease (AD) [5,6]. Lipid profile is a feature insufficiently explored in the area of pathomechanisms of atypical parkinsonisms [7]. In this context, analysis of easily accessible factors in the examination of these entities could seem feasible. Recent studies suggest that high-density lipoprotein (HDL)-derived parameters could be a valuable evaluation tool in differential diagnosis and analysis of pathomechanisms [8].

## 2. Material and Methods

### 2.1. Group Description

This study analyzed biochemical parameters in patients diagnosed with MSA and PSP categorized into three subgroups: parkinsonian variant of MSA (MSA-P), PSP-P and PSP-RS.

The mean age in the MSA-P group was 63.2 years (SD = 8.6), ranging from 51 to 81 years, significantly younger than the PSP-P group (71.1 ± 7.0 years) and the PSP-RS group (72.7 ± 5.8 years). Gender distribution showed a male predominance across all groups: 61.1% in the MSA-P group (7 females, 11 males), 60.0% in the PSP-P group (6 females, 9 males), and 66.7% in the PSP-RS group (5 females, 10 males). The disease duration varied from 3 to 6 years.

All the patients examined in the study were diagnosed in the Department of Neurology of the Medical University of Warsaw by neurologists experienced in movement disorders using the current criteria of diagnosis [1,2]. Participants included in the study provided their written consent. Patients affected by infectious diseases, autoimmune diseases, neoplasmatic diseases, hematologic diseases, dyslipidemia, metabolic syndrome or diabetes or using drugs possibly impacting the examined factors were excluded from the study. Authors excluded patients using drugs possibly affecting the morphological count and lipid profile obtained from the blood sample. All of the participants underwent morphological and biochemical examination in the Department of Laboratory Diagnostics of the Mazovian Brodno Hospital. Authors evaluated non-specific inflammatory parameters commonly discussed in the literature as neutrophil-to-lymphocyte ratio (NLR) and platelet-to-lymphocyte ratio (PLR) and additionally intended to verify the significance of neutrophil, lymphocyte and platelet ratios linked to high-density lipoprotein to assess possible feasibility in the context of the recently highlighted role of lipid profile factor in blood–brain barrier permeability [5,6,7].

### 2.2. Statistical Analysis

All analyses were conducted using Statistica software (version 13.1, Dell Inc., StatSoft, Round Rock, TX, USA). Data distribution was evaluated with the Shapiro–Wilk test. As all parameters followed a normal distribution, results are reported as means with standard deviations (SDs), minimum and maximum values. Group differences were assessed using one-way ANOVA, with Tukey’s HSD test applied for post-hoc pairwise comparisons. Results are illustrated with mean plots and 95% confidence intervals, with *p* < 0.05 considered statistically significant.

## 3. Results

**Figure 1:**
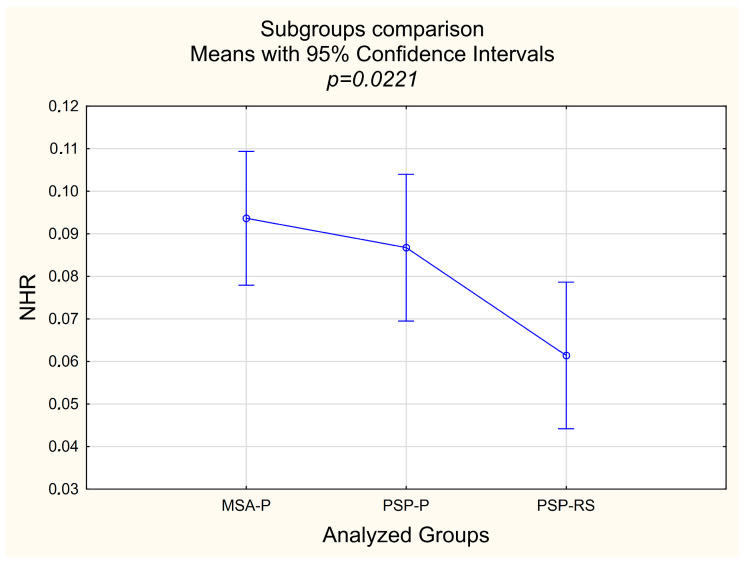
Subgroup comparison. Legend: NHR—neutrophil to high-density lipoprotein ration, MSA-P—Multiple System Atrophy—Parkinsonism Predominant, PSP-P—Progressive Supranuclear Palsy—Parkinsonism Predominant, PSP-RS—Progressive Supranuclear Palsy—Richardson’s Syndrome.

The overall mean age was 68.6 years (SD = 8.4), with significant differences among groups. The MSA-P group had the youngest mean age (63.2 ± 8.6 years), while PSP-P (71.1 ± 7.0 years) and PSP-RS (72.7 ± 5.8 years) represented older cohorts (*p* = 0.0009, Table 1). For biochemical parameters, NLR showed no significant differences (*p* = 0.1166), with overall means ranging from 2.06 (PSP-RS) to 2.80 (MSA-P). Similarly, the PLR demonstrated no significant variation across groups (*p* = 0.6017). However, the neutrophil-to-hemoglobin ratio (NHR) was significantly different (*p* = 0.0221), with the MSA-P group showing the highest mean (0.094 ± 0.028) and the PSP-RS group the lowest (0.061 ± 0.024) [Figure 1]. The lymphocyte-to-hemoglobin ratio (LHR) and platelet-to-hemoglobin ratio (PHR) did not differ significantly among groups (*p* = 0.5879 and *p* = 0.3199, respectively), Table 1.

### Post-Hoc Analysis

Post-hoc analysis using Tukey’s HSD test confirmed significant differences in age and NHR. The MSA-P group’s mean age (63.2 ± 8.6 years) was significantly lower than that of both PSP-P (71.1 ± 7.0 years, *p* = 0.0098) and PSP-RS (72.7 ± 5.8 years, *p* = 0.0016), while no difference was observed between PSP-P and PSP-RS (*p* = 0.8090). For NHR, the MSA-P group (0.094 ± 0.028) had significantly higher values compared to PSP-RS (0.061 ± 0.024, *p* = 0.0211), but no differences were detected between MSA-P and PSP-P (0.087 ± 0.045, *p* = 0.8230) or PSP-P and PSP-RS (*p* = 0.1026) [Figure 1].

## 4. Discussion

The differences between the inflammatory processes in PSP and MSA were not previously widely described. A study by Kwak et al. evaluated inflammatory factors impacted by high-density lipoprotein in PD and Parkinsonism Plus Syndrome (PPS) [9]. The study revealed increased levels of monocyte to high-density lipoprotein cholesterol (MHR) in PD and PPS when compared to controls. Additional subanalysis did not provide significant differences between PD, MSA and PSP. Authors concluded that MHR could be a possibly feasible parameter in the examination of peripherally detected inflammation in the comparison of parkinsonisms and healthy volunteers at early stage of the disease. The study did not acknowledge the subtyping of PSP and the particular indication of PSP-RS and PSP-P. A study on NHR in PSP and CBS showed negative correlation of this parameter with the perfusion in the insula examined using perfusion single photon emission computed tomography only in the CBS group, whereas in PSP, no such observation was detected [7]. The results partly come with the associations between the beneficial role of HDL and the permeability of the blood–brain barrier, which could be crucial in the context of inflammatory aspects of neurodegeneration [5]. Recently performed studies suggest that the inflammatory profile could be a possibly relevant factor in the evaluation of the two entities. Analyzes on microglial-derived interleukins (Interleukins 1β and 6) and hepcidin in PSP-P and PSP-RS examined at the same stages revealed higher levels of interleukins in PSP-P in the CSF and serum and higher levels of hepcidin in PSP-RS [10,11]. The interleukins were found to be possibly protective in the context of atrophic changes observed in the course of the pathogenesis of PSP [12]. Moreover, additional analyses showed negative correlation between the levels of NLR and PLR and Montreal Cognitive Assessment scale [13]. The same study showed weak negative correlation between Interleukin 1β and MoCA. The works analyzing the significance of other interleukins did not provide conclusive results. An examination of interferon γ, Interleukin 10, Interleukin 18, Interleukin 1β, Interleukin 4, Interleukin 6, transforming growth factor β1, and TNF-α showed increased levels of microglial-derived interleukins in PSP and MSA when compared to PD [14]. Other studies on the significance of inflammatory factors in PSP revealed ambiguous results. Neutrophil-to-lymphocyte ratio was found to have higher values in PSP when compared to PD and controls [15]. A meta-analysis on peripheral immune profile and NLR showed increased levels in PD and PSP when compared to controls. Moreover, it was concluded that the inflammation in PSP could be linked the neutrophil activation [16].

The profile of peripheral inflammatory factors in MSA was not extensively studied. An evaluation of the levels of NLR and PLR in MSA and PD showed increased levels of NLR and PLR in the comparison with control groups; however, in MSA, the observation was confirmed only for NLR [17]. It was concluded that the stimulation of neutrophils in MSA may be impacted by mechanisms related to GSK3β inhibition [18]. The peripheral inflammatory aspect of MSA was analyzed in the context of MHR; in one of the studies, its levels were found to be higher than in PD [19]. It was also revealed that MSA may be linked with increased levels of NLR and red cell distribution width to platelet ratio. Other studies based on the analysis of IL-1β, Interleukin 2, IL-6, Interleukin-10, tumor necrosis factor-α and high sensitivity C-reactive protein in the serum did not show any significant differences between MSA and controls. It was stressed that the levels of peripheral inflammatory factors may come up with the disease duration [20].

The results obtained from this initial study confronted with the contemporary literature presented above suggests that the peripheral inflammatory profile of atypical parkinsonisms should be interpreted as a likely multifactorial process. The results obtained from patients with PSP-P, PSP-RS and MSA-P in this study suggest that the disease duration and possible pathological background are not necessarily the two major determinants of inflammatory manifestation. The most striking issue is related to the heterogeneity within the PSP group. The less pronounced clinical deterioration in PSP-P comes up with an increased level of NHR compared to that in PSP-RS, which could lead to various hypotheses. The rapid growth of inflammatory factors in PSP-RS may be present at an initial stage, which could explain why in the more advanced periods of PSP-P, the levels are higher than in PSP-RS. This study revealed significant differences between MSA-P and PSP-RS only in NHR, which could lead to the point that inflammation may have a relevant difference in the context of neutrophil activation, which was not detected in the other parameters examined in this study. The role of HDL in the inflammatory process requires additional analyses using other morphological parameters impacted by this factor.

## 5. Limitations

The study is affected by several limitations. Due to the pilot character of the study, the number of examined patients ranges between 15 and 18 patients depending on the group. No additional control group was added; authors referred to physiological ranges used by the Laboratory Diagnostics Department. This was related to the fact that the evaluation was partly conducted during the COVID-19 pandemic, which made it difficult to obtain a sufficient group of healthy volunteers. The groups of PSP patients are older than patients with MSA, which is related to the fact that the age of initiation of symptoms in MSA is lower than in PSP; the authors intended to examine patients with matched disease durations. Due to the fact that all of the examined patients are alive, no neuropathological verification was performed, and the study is based on probable or clinically established diagnoses. Moreover, the study is based on a single evaluation which excludes the possibility of analyzing tendencies in non-specific peripheral inflammation. The study is based on the analysis of serum without analysis of other human fluid samples. The authors intended to perform an examination which could be easily accessible and minimally invasive.

## 6. Conclusions

The inflammatory profiles of atypical parkinsonisms differ. The parameters evaluated in the study suggest that the differences of the inflammatory process may not necessarily be present at the boundaries of synucleinopathic and tauopathic atypical parkinsonisms, as the profiles of MSA-P and PSP-P did not show significant differences. The study stresses the issue that examination of the pathomechanism in PSP should be performed with the acknowledgement of two major subtypes of PSP. To the best of our knowledge, this is the first study providing a preliminary evaluation of PSP-P using peripheral inflammatory parameters impacted by HDL. Due to the limitations of the study, more research in the field is inevitably required.

## Figures and Tables

**Table 1:** Descriptive statistics and subgroup comparison.

	ALL (N = 48) (F/M = 24/24)				MSA-P (N = 18) (F/M = 10/8)				PSP-P (N = 15) (F/M = 9/6)				PSP-RS (N = 15) (F/M = 5/10)				
	Mean	Min	Max	SD	Mean	Min	Max	SD	Mean	Min	Max	SD	Mean	Min	Max	SD	*p*
Age	68.6	51.0	83.0	8.4	63.2	51.0	81.0	8.6	71.1	61.0	81.0	7.0	72.7	62.0	83.0	5.8	0.0009
NLR	2.43	0.79	5.37	1.05	2.80	0.80	5.37	1.22	2.34	1.44	4.10	0.85	2.06	0.79	4.48	0.90	0.1166
PLR	147.8	46.6	321.1	52.0	137.9	72.2	242.6	46.2	154.5	98.9	321.1	59.1	153.1	46.6	231.1	52.7	0.6017
NHR	0.081	0.022	0.207	0.035	0.094	0.030	0.153	0.028	0.087	0.022	0.207	0.045	0.061	0.026	0.098	0.024	0.0221
LHR	0.037	0.014	0.106	0.019	0.037	0.018	0.062	0.013	0.041	0.014	0.106	0.026	0.033	0.015	0.073	0.017	0.5879
PHR	5.0	1.9	12.0	2.1	4.8	2.9	8.3	1.5	5.6	1.9	12.0	2.9	4.5	2.1	7.2	1.5	0.3199

Legend: *p*—*p* value for one-way ANOVA. Legend: NLR—neutrophil-to-lymphocyte ratio, PLR—platelet-to-lymphocyte ratio, NHR—neutrophil to high-density lipoprotein ratio, LHR—lymphocyte to high-density lipoprotein ratio, PHR—platelet to high-density lipoprotein ratio, MSA-P—Multiple System Atrophy—Parkinsonism Predominant, PSP-P—Progressive Supranuclear Palsy—Parkinsonism Predominant, PSP-RS—Progressive Supranuclear Palsy—Richardson’s Syndrome.

## Data Availability

The original contributions presented in this study are included in the article. Further inquiries can be directed to the corresponding author.
